# Supplementary material for: Impact of the 13-Valent Pneumococcal Conjugate Vaccine on Severe Invasive Disease Caused by Serotype 3 Streptococcus Pneumoniae in Italian Children
Source: Vaccines (Basel). 2019 Sep 24;7(4):128. doi: 10.3390/vaccines7040128 (PMC6963450; doi:10.3390/vaccines7040128)
Supplement: Supplementary file 1 [file vaccines-07-00128-s001.pdf]

SUPPLEMENTARY FILE

Table S1. Mean vaccination coverage for PCV13 in Italy.

Year	Italian mean coverage	Minimum-maximum (Regional data)
2012	Data not available for all Italian Regions	Data not available for all Italian Regions
2013	86.9	44.5–95.9*
2014	87.5	76.6–98.5
2015	88.8	81.0–92.8
2016	88.3	80.5–97.0
2017	90.9	83.8–96.7

*only 3/20 Italian Regions: Calabria, Abruzzo and Sardegna (which together account for 7.6% of Italian population 0–16 years) were below 75% coverage for Streptococcus pneumoniae vaccination.
Available online: http://www.salute.gov.it/portale/documentazione/p6_2_8_3_1.jsp?lingua=italiano&id=20 (accessed on 18 July 2019).

Table S2. Person years for each age class and each study year during the study. Data were obtained from the National Italian Statistic Institute (ISTAT) at http://demo.istat.it/. The pre-PCV13 period is shown in Yellow is shown and the post-PCV13 period in blue. Total "million person years" have been calculated and used as denominators for crude incidence rates.

	**Year**	**2006**	**2007**	**2008**	**2009**	**2010**	**2011**	**2012**	**2013**	**2014**	**2015**	**2016**	**2017**	**2018 Jan-Aug**	**Total Million Person Year**		
Age Class															Age Class	Pre PCV13	Post PCV13
7–8		542,089	548,265	562,861	559,967	561,260	568,432	561,023	559,948	573,979	574,211	576,106	569,379	326,656	7–8	6,757,520	326,656
6–7		543,966	557,310	555,705	558,416	566,434	575,185	556,531	563,851	574,555	576,926	570,072	560,438	317,865	6–7	6,198,951	878,303
5–6		552,603	550,057	554,741	563,618	572,851	569,347	560,414	563,733	576,775	570,721	560,984	545,362	311,765	5–6	5,634,860	1,418,111
4–5		545,498	549,414	559,960	569,655	567,113	573,500	560,099	565,676	570,547	561,808	546,008	534,694	298,638	4–5	5,061,462	1,941,148
3–4		545,108	554,812	565,975	563,816	570,925	572,270	561,250	559,136	561,406	546,910	534,890	511,743	293,417	3–4	4,493,292	2,448,366
2–3		550,865	561,735	559,638	567,379	569,438	577,278	554,608	549,886	546,986	535,706	511,760	502,078	284,054	2–3	3,940,941	2,930,470
1–2		557,219	554,884	563,429	566,368	574,611	567,607	544,814	538,422	536,114	511,794	500,679	485,283	276,247	1–2	3,384,118	3,393,353
0–1		547,159	554,966	558,624	569,883	562,566	555,791	531,572	524,021	509,053	496,627	479,611	467,657	266,112	0–1	2,793,198	3,830,244

Table S3. Serotype-specific invasive pneumococcal disease number of cases and intragroup percentage representation in Italian children under 8 years of age born before 2011, after 2011 and vaccinated with PCV13. Non-PCV13: Pneumococcal serotypes not included in PCV13.

Serotype	Cases in Born before 2011	Cases in Born after 2011	Cases in Born after 2011 and Vaccinated	% in Born before 2011	% in Born after 2011	% in Vaccinated Children
1	61	2	2	28.24	2.94	5.26
3	38	22	16	17.59	32.35	42.11
4	1	0	0	0.46	0.00	0.00
5	6	0	0	2.78	0.00	0.00
6/6A/6B	11	1	0	5.09	1.47	0.00
7F	17	1	0	7.87	1.47	0.00
9V	1	1	0	0.46	1.47	0.00
14/14B	8	1	0	3.70	1.47	0.00
18	2	1	1	0.93	1.47	2.63
19A	30	3	0	13.89	4.41	0.00
19F/B	6	1	0	2.78	1.47	0.00
23F	2	0	0	0.93	0.00	0.00
Non-PCV13	33	35	19	15.28	51.48	50.00
Total	216	68	38	100	100	100

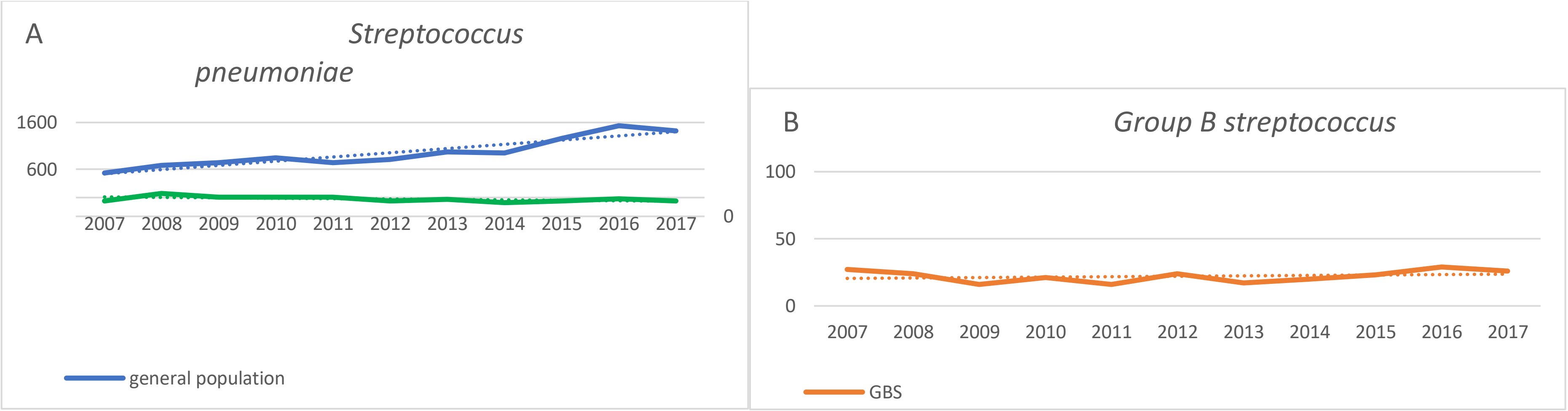

Figure S1. Influence of the surveillance of invasive bacterial diseases (IBD) in Italy. Ministerial data on number of cases per year of invasive bacterial diseases caused by *Streptococcus pneumoni*ae (**A** – continuous blue line) and by Group B Streptococcus (**B**) from 2007 to 2017 in Italy. Starting from 2015 the registered number of cases of *Streptococcus pneumoniae* (SP) IBD in the general population has progressively increased. Based on data released by Italian Health Institute, such a raise is dependent on the improvement in diagnostic sensitivity and on the increase in attention paid by health professionals to surveillance of *Neisseria meningitidis, Streptococcus pneumoniae* and *Haemophilus influenzae* all over Italy in consequence of specific projects of the Italian Ministry of Health. Cases due to pathogens not subject to surveillance - such as *Group B streptococci* (GBS) - did not increase in the last years. Panel A also shows the number of cases of IBD caused by *Streptococcus pneumoni*ae in children under 9 years of age (continuous green line) that is substantially stable despite the effect of the augmented surveillance. Such a trend is likely due to the impact of the pneumococcal conjugate vaccination (PCV) which has been offered to this age group. Dotted lines represent trends.

The "Surveillance of invasive bacterial diseases in Italy" is a project of the Italian Health Institute (Italian Ministry of Health) and works in parallel but separately from the Molecular Surveillance Registry. Available online: http://old.iss.it/binary/mabi/cont/Report_MBI2_2007_2010.pdf; http://old.iss.it/binary/mabi/cont/Report_MBI_20150812_V5.pdf; http://old.iss.it/binary/mabi/cont/Report2017.pdf; (accessed on 18 July 2019).
